# Correction: Seed germination ecology of *Conyza stricta* Willd. and implications for management

**DOI:** 10.1371/journal.pone.0248083

**Published:** 2021-02-26

**Authors:** Safdar Ali, Fakhar Din Khan, Rehmat Ullah, Rahmat Ullah Shah, Saud Alamri, Maeesh AlHarthi, Manzer H. Siddiqui

In the Funding statement, the RSP number from the funder Researchers Supporting Project, King Saud University, Riyadh, Saudi Arabia is listed incorrectly. The correct RSP number is: RSP-2020/194.
